# An Innovative Use of Point-of-Care Ultrasound for Identification and Management of a Rare and Uncommon Perioperative Complication: Periorbital Emphysema Following Sinus Surgery in a Pediatric Patient

**DOI:** 10.7759/cureus.57721

**Published:** 2024-04-06

**Authors:** Jyoti Kanwat, Gopal Jalwal, Uma Rathi, Kuljeet Kaur

**Affiliations:** 1 Anaesthesiology and Critical Sciences, All India Institute of Medical Sciences, Bathinda, Bathinda, IND; 2 Anaesthesiology, All India Institute of Medical Sciences, Bathinda, Bathinda, IND; 3 Anaesthesiology and Intensive Care, All India Institute of Medical Sciences, Bathinda, Bathinda, IND

**Keywords:** lamina papyracea, laryngospasm, pocus in anesthesiology, periorbital emphysema, functional endoscopic sinus surgery (fess)

## Abstract

Periorbital emphysema is a rare complication following functional endoscopic sinus surgery (FESS) with potential sight-threatening consequences. We present a case of an eight-year-old male who developed periorbital emphysema after FESS for allergic fungal sinusitis. Prompt diagnosis was made using point-of-care ultrasound (POCUS), facilitating timely intervention and conservative management. This case underscores the importance of perioperative imaging to identify lamina papyracea abnormalities, smooth extubation to prevent complications, and the innovative use of POCUS in diagnosing perioperative orbital emphysema and managing it conservatively while examining the eye at regular intervals. These findings highlight the significance of vigilance during FESS procedures and the utility of POCUS in diagnosing and managing rare perioperative complications.

## Introduction

Functional endoscopic sinus surgery (FESS) procedure in the management of various sinus and orbital pathologies, offers significant therapeutic benefits to patients. However, despite advancements in surgical techniques and perioperative care, these procedures can be associated with rare but potentially severe complications such as periorbital emphysema and optic nerve injury. Although these complications typically resolve on their own, there is a risk that they may spread and result in permanent blindness [1].

Computed tomography is widely considered the optimal diagnostic method for periorbital emphysema. However, its use during surgery is not always practical, and there is the risk of radiation exposure associated with this imaging technique [2]. In such cases, point-of-care ultrasound (POCUS) can be a valuable alternative, as it not only allows for diagnosing periorbital emphysema but can also be performed repeatedly to monitor its resolution. Herein, we present a case report of periorbital emphysema detailing the occurrence, diagnosis with point of care ultrasound, and management of this complication in an eight-year-old male undergoing FESS secondary to allergic fungal sinusitis.

## Case presentation

An eight-year-old male weighing 32 kg presented with a complaint of left-side orbital proptosis secondary to allergic fungal sinusitis for the past six months. The patient was scheduled for left-side FESS and orbital decompression under general anaesthesia. There was no history of recent upper airway infection or other chronic illnesses. A preoperative CT scan revealed fungal involvement of the left side nasal cavity, maxillary sinus, ethmoidal sinus, sphenoid sinus and bilateral frontal sinus.

After informed and written consent from parents, as well as fasting confirmation, the patient was shifted to the operation theatre (OT). After premedication with glycopyrrolate (.005mg/kg), midazolam (.02mg/kg) and fentanyl (2mcg/kg), the patient’s induction was performed using propofol (2mg/kg) and vecuronium (0.1mg/kg) then the trachea was intubated with a 5.5-cuffed endotracheal tube confirming bilateral equal air entry. Anaesthesia was maintained on an oxygen-air gas mixture with sevoflurane and intermittent muscle relaxant doses. A throat pack was introduced before starting surgery to prevent blood from trickling into the larynx and oesophagus. During the surgical procedure, the left lamina papyracea was identified and excised, which was infected with a fungal mass. At the completion of the surgery, nasal packing was done with merocel in both nostrils.

**Figure 1:**
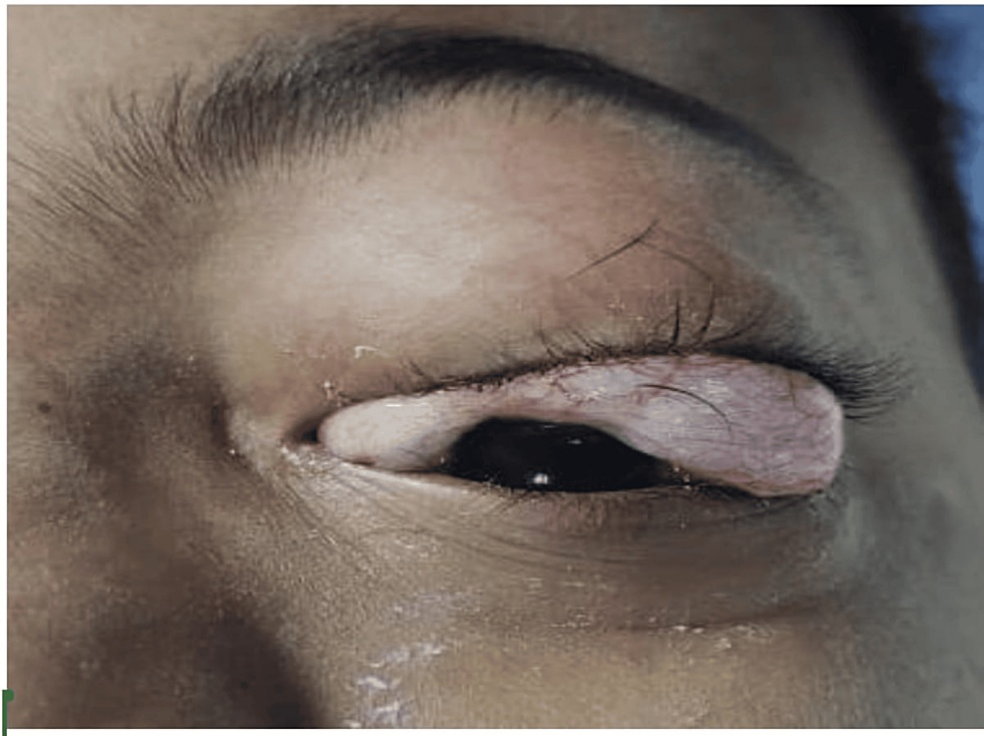
Periorbital emphysema

The throat pack was fully soaked with blood, and it was removed after proper suctioning of the airway. After adequate reversal of neuromuscular blockade, considering the smooth extubation, the endotracheal tube was taken out after proper oral suctioning and once at an acceptable tidal volume, and full consciousness was achieved. Unfortunately, the patient experienced laryngospasm immediately after being extubated, resulting in a decline in oxygen saturation, for which propofol, positive pressure bag and mask ventilation were given. After a while, the laryngospasm subsided but left periorbital emphysema (Figure 1) developed, which was progressive with positive pressure mask ventilation. The patient was again intubated after being given muscle relaxant (vecuronium) and propofol, and nasal endoscopy was performed to rule out any bleeding as well as the cause of periorbital emphysema but could not detect anything.

**Figure 2:**
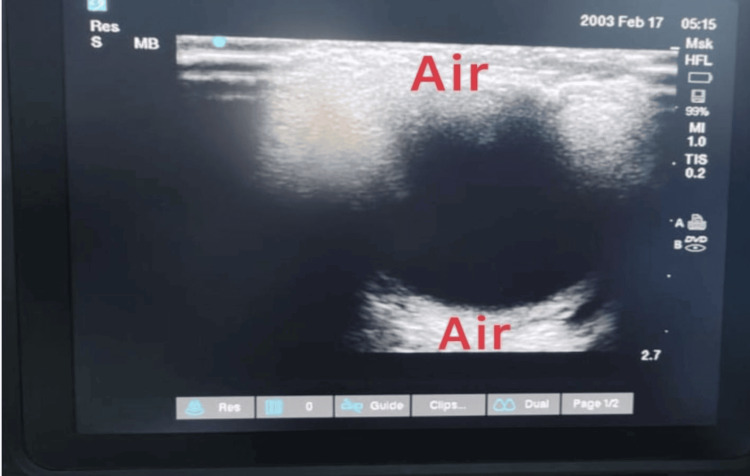
Point-of-care ultrasound (POCUS) showing air around the eyeball confirming the diagnosis of periorbital emphysema

The ophthalmologist found normal orbital pressure and intact pupillary reflexes and ruled out any retro-orbital hematoma. The diagnosis of orbital emphysema was confirmed by point-of-care ultrasound, revealing a pocket of air around the eyeball (Figure 2). As a conservative treatment, Neosporin ointment was applied to the patient's eye, and a pressure bandage was placed over it. To reduce airway reactivity, levosalbutamol puffs were administered. Finally, after gentle airway suction and muscle relaxant reversal, the patient was again extubated after 30 minutes then the patient was shifted to the ICU for observation, where intermittent bronchodilator with steroid nebulization continued for one day. The patient was followed up by an ophthalmologist (assessing IOP, vision, and swelling of emphysema ) and all findings were within normal range. Within 12 hours, the swelling started to recede, and it went off completely after 36 hours. It was advised to the patient to avoid any coughing and nasal blowing postoperatively. On fundoscopy, the posterior compartment was normal, the vision of the eye was 6/6, and the patient did not show any delayed sequelae of eye complication.

## Discussion

The presented case underscores the importance of prompt recognition and management of periorbital emphysema complications in pediatric FESS. Periorbital emphysema, though rare, can occur during or after these procedures and require prompt intervention to prevent potential adverse outcomes.

Orbital complications during FESS are rare but can be serious when they occur. The orbit is separated from the nasal cavity by a thin bone called the lamina papyracea. The breach of the lamina papyracea during surgery can result in periorbital emphysema, a rare complication characterized by the infiltration of air into the periorbital tissues. While the exact mechanism of air ingress remains speculative, it is likely attributed to positive pressure ventilation during surgery, leading to air tracking from the nasal cavity or sinuses into the periorbital space through the breached bony barrier [3, 4].

While a small amount of periorbital air is not unheard of during FESS, clinically significant periorbital emphysema, implies that the condition is causing notable symptoms or complications that require medical attention, is an uncommon but serious complication that, if left untreated, can lead to irreversible blindness and other catastrophic consequences. It is essential to manage this condition properly to prevent such severe outcomes [5].

CT scan is a reliable method for determining the presence and location of air in orbital emphysema cases. It can accurately identify bony defects but feasibility and radiation exposure in the pediatric population is a major drawback [3]. Magnetic resonance imaging (MRI) may be a valuable tool for further assessing soft tissue pathology in the rectus muscles, as well as evaluating the optic nerve and brain, and detecting vascular damage [6]. POCUS is a beneficial diagnostic tool for identifying various global defects, such as the presence of foreign bodies, dislocation of lenses and retinal pathology [7].

 In our case, lamina papyracea was infected by a fungal infection and had to be removed during the surgical procedure. However, despite taking all necessary precautions for smooth extubation, an unexpected laryngospasm triggered clinically significant periorbital emphysema, which continued to worsen with positive pressure ventilation. In the outpatient setting, we usually transfer the patient to the CT scan room to confirm the diagnosis, but this was not feasible in the operating room. Therefore, the timely use of POCUS provided an accurate diagnosis and guided conservative management, highlighting the importance of this modality in perioperative settings.

Orbital emphysemas are managed primarily based on clinical signs and symptoms and imaging findings. Clinical manifestations of orbital emphysema can include eye pain, decreased sensation, limited ocular motility or enophthalmos, double vision, and vision loss. The management plan will vary depending on the severity and location of the fracture or emphysema. It is important to carefully evaluate the patient's symptoms and imaging results to determine the appropriate course of action [8]. In our case, an ophthalmologist diagnosed clinically significant orbital emphysema using POCUS. However, there were no signs of vision loss, and the pupillary reflexes remained intact. Fortunately, the condition resolved on its own within a few days, without any long-term consequences or complications.

## Conclusions

This article highlights a unique application of POCUS in diagnosing perioperative orbital emphysema and distinguishing it from other causes of periorbital swelling. Patients who present with bony defects in the lamina papyracea or necessitate their extraction during surgery must be promptly identified. It is imperative to avert situations that may result in laryngospasm and positive pressure ventilation. Additionally, prompt diagnosis and management of periorbital emphysema are of utmost importance to avert any further ocular complications.
